# Method for Valorization of Coffee Cherry Waste via Hydrothermal Valorization Using Organic and Inorganic Acids as Catalysts

**DOI:** 10.3390/mps7060087

**Published:** 2024-10-29

**Authors:** Alejandra Sophia Lozano Pérez, Valentina Romero Mahecha, Carlos Alberto Guerrero Fajardo

**Affiliations:** Departamento de Química, Universidad Nacional de Colombia, Carrera 30 No. 45-02 Ed., Bogotá 111321, Colombia

**Keywords:** platform chemical, hydrothermal valorization, homogeneous catalysts, coffee cherry waste

## Abstract

The valorization of coffee cherry waste through hydrothermal carbonization (HTC) was investigated using various organic and inorganic acid catalysts to produce platform chemicals. This study aimed to evaluate the effectiveness of these catalysts for enhancing reaction rates, improving yields, and promoting selectivity. The results showed that sulfuric acid and adipic acid were the most effective, each resulting in a 20% increase in the total yield, demonstrating the potential of organic acids as efficient catalysts in HTC. Other catalysts, such as benzoic acid and phenylacetic acid, also showed promising results, while butyric acid significantly decreased the total yield. The most abundantly produced platform chemicals were sugars, followed by formic acid, levulinic acid, HMF, and furfural. These findings highlight the potential of coffee cherry waste as a valuable resource for producing key chemicals, and the feasibility of hydrothermal carbonization as a sustainable approach for biomass valorization. This study emphasizes the importance of selecting the appropriate catalysts to optimize the conversion process and maximize the extraction of valuable chemicals. The environmental and economic implications of these findings are significant, as they can contribute to the development of sustainable and efficient biomass utilization technologies that could transform agricultural waste into high-value products while reducing waste and promoting a circular economy.

## 1. Introduction

Hydrothermal processes are increasingly recognized for their potential to valorize agricultural waste by converting biomass into valuable products, such as biofuels, chemicals, and materials [1]. These processes utilize high-temperature water in a closed system, enabling chemical reactions that break down complex organic materials [2]. The ability to operate above the boiling point of water enhances the solubility and reactivity of both organic and inorganic compounds, making hydrothermal treatment an efficient and effective method for biomass conversion. This approach not only addresses the challenge of agricultural waste disposal, but also contributes to the development of sustainable resources [3].

The optimization of the variables within hydrothermal processes is critical for maximizing the efficiency and effectiveness of biomass valorization. The key parameters, including temperature, pressure, reaction time, and pH, significantly influence the reaction kinetics and the types of products generated [4]. For instance, higher temperatures can accelerate reaction rates, leading to increased yields of the desired products; however, they may also risk the degradation of sensitive compounds. By systematically adjusting these variables, researchers can tailor the hydrothermal treatment to enhance product purity and yield, thereby improving the overall feasibility of the process [5].

Ultimately, the significance of hydrothermal processes and their optimization lies in their dual benefit of promoting environmental sustainability while creating economic opportunities [6]. As the global demand for renewable resources continues to rise, effective hydrothermal methods could transform agricultural waste into high-value products, contributing to a circular economy [7]. This innovative approach could not only mitigate waste disposal challenges but also support the transition to more sustainable agricultural practices, making it a vital area of research and development in the quest for greener technologies [8].

The use of low temperatures in hydrothermal processes, combined with the application of catalysts, is crucial for enhancing the efficiency of biomass conversion while minimizing energy consumption [9]. Operating at lower temperatures not only reduces the energy requirements of the process, but also mitigates the degradation of sensitive organic compounds, preserving valuable materials that can be extracted from agricultural waste [10]. By leveraging catalysts, particularly homogeneous catalysts, the reaction kinetics can be significantly improved, allowing for effective biomass conversion even under milder conditions. This approach aligns with the principles of green chemistry, promoting sustainability and reducing the environmental impact of waste management [11].

Water is an essential component of hydrothermal processes, which convert lignocellulosic biomass into platform chemicals by dissolving complicated biomass structures at high pressures and temperatures [12]. The hydrolysis of cellulose and hemicellulose releases individual sugar monomers, such as glucose and xylose [13]. Following that, these sugars can undergo further reactions, including dehydration, to produce furfural and 5-hydroxymethylfurfural (HMF), which are platform chemicals. Lignocellulosic biomass can be hydrolyzed effectively due to the unique properties of water at high pressures and temperatures, particularly the increased concentration of H^+^ ions [14].

Homogeneous catalysts, which exist in the same phase as the reactants, are particularly advantageous for hydrothermal processes due to their ability to facilitate reactions without requiring extensive modifications to the existing process conditions [15]. Common homogeneous catalysts used in these processes include acids, bases, and metal salts. For instance, sulfuric acid and hydrochloric acid can be employed to hydrolyze cellulose and hemicellulose, enhancing the yield of fermentable sugars [16]. Additionally, metal salts, such as zinc chloride and sodium carbonate, can act as catalysts to promote the conversion of biomass into valuable chemicals. The use of these catalysts allows for the optimization of reaction pathways, leading to higher product yields and selectivity [17].

The efficiency of using organic and inorganic acids as catalysts in hydrothermal processes varies significantly, based on their chemical properties and the specific reactions involved. Organic acids, such as acetic and formic acid, are often favored for their ability to enhance product yields and improve the overall reaction environment [18]. These acids can stabilize reaction intermediates and facilitate the breakdown of complex biomolecules at lower temperatures compared to inorganic acids. For example, studies have shown that organic acids can generate higher pressures during hydrothermal liquefaction, leading to increased yields of gaseous products, like methane and hydrogen, which are valuable for energy recovery [19]. Moreover, organic acids tend to produce bio-crude of better quality, although they may not always enhance the heating value of the product [20]. The range of organic and inorganic acids currently utilized as catalysts in hydrothermal processes is limited, highlighting a significant research gap that warrants further investigation. Expanding the variety of homogeneous catalysts could enhance the effectiveness and versatility of hydrothermal carbonization, leading to improved biomass conversion outcomes. Addressing this gap is essential for diversifying the catalyst options and optimizing the hydrothermal processes for a broader range of applications.

In contrast, inorganic acids, such as sulfuric acid, are known for their strong catalytic activity due to their ability to liberate a high number of hydronium ions, which can significantly accelerate hydrolysis reactions [21]. However, the use of inorganic acids can lead to more corrosive environments and may negatively impact the quality of the bio-crude produced. While they can effectively hydrolyze biomass and improve the yield of fermentable sugars, they may also result in the formation of undesirable by-products and a lower heating value of the final product. The choice between organic and inorganic acids often depends on the desired product and process conditions, with organic acids generally providing a more favorable reaction environment for biomass conversion [22].

The primary aim of this article is to develop a method for maximizing the extraction of platform chemicals from coffee cherry waste (an important waste that is not managed and has significant amounts of cellulose and hemicellulose to be valorized) through hydrothermal carbonization (HTC), while evaluating the effectiveness of various organic and inorganic acid catalysts. By optimizing the hydrothermal carbonization process and strategically selecting suitable catalysts, this study seeks to enhance the yield, efficiency, and specificity of the target platform chemicals derived from this abundant agricultural waste stream [22]. This research, which systematically investigates the parameters of hydrothermal carbonization and the selection of catalysts, contributes to the expanding field of biomass conversion, promoting the development of green technologies that can effectively transform agricultural waste into high-value products while minimizing energy consumption and reducing the environmental impact.

## 2. Materials and Methods

### 2.1. Characterization of the Biomass of the Coffee Cherry Waste Samples

Coffee cherry waste samples were collected from a farm located in Santandercito, Cundinamarca, Colombia. The samples were ground to a particle size of 1 mm, identified as optimal in previous research [23], following a drying process to achieve a consistent weight. To determine the conversion of the initial components into the desired products, it was essential to determine the composition of the starting biomass. Using the method outlined in “Methods for Dietary Fiber, Neutral Detergent Fiber, and Non-starch Polysaccharides in Relation to Animal Nutrition” in the *Journal of Dairy Science* [23], the initial chemical composition (lignin, hemicellulose, and cellulose) was determined.

### 2.2. Biomass Hydrothermal Reaction

The tests were conducted in a 500 mL reactor without stirring, at 180 °C, with a biomass particle size of 1 mm, because of previous screenings that showed that these conditions are ideal for the production of sugars and platform chemicals, without leading to high amounts of degradation and hydrochar production [24]. Once the reactor temperature reached 180 °C, the reaction was carried out for one hour. A total of 5 g of biomass and 95 g of catalyst solution (1:20 biomass-to-solution ratio) were added to the reactor. Previous research showed that a 1:20 ratio was the best ratio for platform chemical extraction from biomass [25]. The concentration of the acid solutions was 0.02 M; this was chosen in order for it to be strong enough to catalyze the reaction, but not so concentrated that it could harm the column in the HPLC-RI process.

To investigate the effects of catalysts on the hydrothermal process, a few homogeneous acid catalysts were used. The set of inorganic acids used included sulfuric acid, hydrochloric acid, nitric acid, and phosphoric acid. The organic acids employed were anthranilic acid, 4-aminobenzoic acid, chloroacetic acid, phthalic acid, benzoic acid, phenylacetic acid, adipic acid, butyric acid, propanoic acid, and succinic acid.

The selection of both organic and inorganic acids for this study is justified by their diverse pKa values, functional groups, and the roles they play in influencing chemical processes. The inorganic acids chosen—sulfuric acid, hydrochloric acid, nitric acid, and phosphoric acid—exhibit strong acidity with pKa values typically below 1, making them effective in promoting hydrolysis and facilitating various chemical reactions. Their high acidity allows for significant proton donation. In contrast, the organic acids exhibit a broader range of pKa values, from approximately 2.87 for chloroacetic acid to around 5.6 for succinic acid. This variability in acidity and the presence of different functional groups (carboxylic, amine, and aromatic groups) allow for a comprehensive examination of how these acids interact with substrates and influence processes such as hydrothermal treatment and bioleaching. The combination of strong inorganic acids and a variety of organic acids provides a robust framework with which to study their effects on chemical transformations, thus enhancing the overall methodology of the research.

### 2.3. Characterization of Liquid and Solid Fractions Obtained

After one hour of reaction, the reactor was cooled, and the product was filtered. After the recovery of the liquid phase, the conductivity and pH were measured to track the reactions that were taking place. Furthermore, the platform chemicals (PC) underwent HPLC-RI quantification. The yields were calculated using Equation (1).

(1) $$\%\;{Yield}_{PC}=\frac{\left(grams\;of\;platform\;chemicals\;obtained\right)}{\left(grams\;of\;lignocelullosic\;structure\;in\;biomass\right)}\times 100\%$$

**Figure 1:**
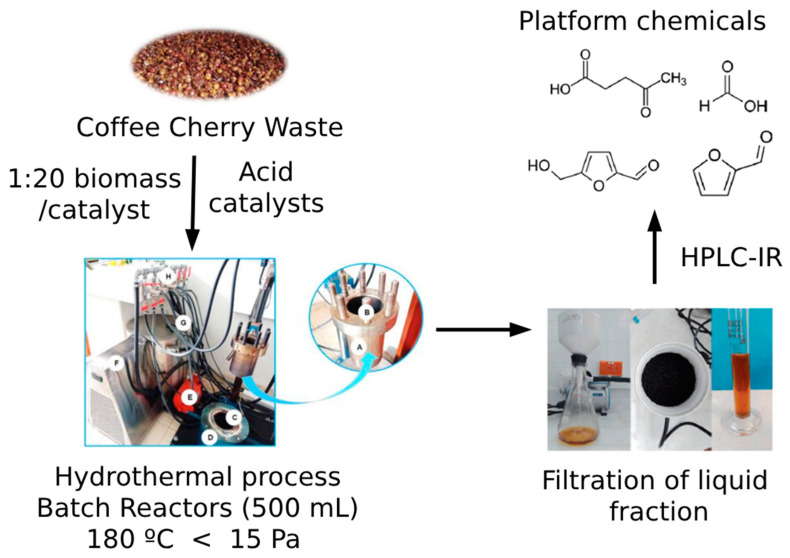
Method for the extraction of platform chemicals from coffee cherry waste using hydrothermal carbonization (HTC) in a batch reactor for 1 h at 180 °C and 15 Pa. The implemented reactor consists of a jacket (A), eight threaded studs (B) in the upper part; a heating blanket allows for the regulation of the reaction temperature (C and D), and the coil is used for the circulation of water to stabilize the temperature with the operation of the chiller (F) and its respective pump (E). The water faucets (H) allow for a control of the water recirculation rate which is pumped through the hoses (G).

The solid fraction was dried at 105 °C until it attained a constant weight and was measured to calculate the matter balance (Figure 1).

### 2.4. Quantification of Produced Platform Chemicals

**Figure 2:**
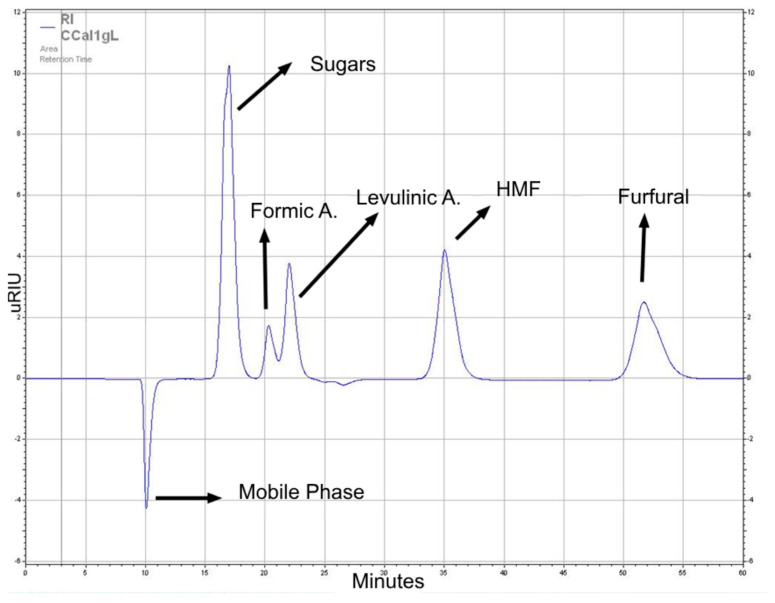
Chromatogram of standards used to identify and quantify platform chemicals obtained.

The methodology used in previous research was employed for the platform chemicals (PC) quantification. The presented method was carried out employing a Hitachi Elite LaChrom (Tokyo, Japan), a Hitachi L-2490 refraction index detector at 40 °C, a SHODEX Sugar SH1821 column at 60 °C, 0.005 M H_2_SO_4_ for the mobile phase, and a flow rate of 0.5 mL/min. Standards of formic acid, furfural, xylose, glucose, mannose, arabinose, HMF, and levulinic acid (Sigma-Aldrich—St. Louis and Burlington, MA, USA) were utilized to determine the platform chemicals’ retention times (Figure 2). The different sugars appear at the same retention time, and this is why they are quantified and identified as a group of sugars. The calibration curves were designed with multi-standard solutions between 0.1 and 10 g/L in order to measure a large range of concentrations, which were prepared and measured 5 times to ensure exactitude and repeatability. “Others” are components that were not identified, either because of the lack of standards with which to quantify them, or because they were components from the initial biomass, such as lipids, fiber, tannins, caffeine, and phenols, that were not transformed and were not of much interest.

## 3. Results and Discussion

Coffee cherry residues, a by-product of coffee production, are a waste with the potential to be used as a raw material in hydrothermal processes. A preliminary biomass characterization (Table 1) was performed to identify the amount of lignin, cellulose, and hemicellulose present in the waste. The composition of the biomass was 13.7% lignin, 12.5% hemicellulose, and 27.6% cellulose. Coffee cherry residues can be effectively utilized to develop bioprocesses aimed at the sustainable production of chemical products. This is made possible by harnessing the valuable components found in these residues, including lignin, cellulose, and hemicellulose. Lignin, a complex organic polymer, can serve as a precursor for biofuels and other aromatic compounds, while cellulose and hemicellulose can be converted into fermentable sugars. These sugars can then be further processed through microbial fermentation or enzymatic hydrolysis to produce a variety of high-value chemicals and bio-based materials. By leveraging the inherent properties of coffee cherry residues, we can create environmentally friendly and economically viable pathways for chemical production, thereby contributing to waste reduction and promoting a circular economy in the agricultural sector [26].

Coffee cherry waste exhibits a distinctive composition that influences its potential for valorization and conversion into valuable products. The elemental analysis revealed a carbon (C) content of 45.27%, which is significant for energy production and carbon-based material synthesis. The hydrogen (H) content of 4.862% contributes to the energy yield during combustion or pyrolysis processes. Additionally, the nitrogen (N) content of 1.471% indicates the presence of proteins and amino acids, which may enhance the nutritional profile of the biomass or serve as precursors for biofertilizers. The sulfur (S) content of 0.138% is relatively low, minimizing potential emissions during thermochemical processes. The initial moisture content of the coffee cherry waste is notably high at 80.79%, which poses challenges for processing, but can be reduced to 10.94% through air-drying to obtain the biomass used in hydrothermal processes (BHP). The waste also contains 7.79% ashes, 10.06% volatile matter, and 1.36% fixed carbon, indicating its suitability for various valorization processes.

The valorization of coffee cherry residues through hydrothermal processes not only provides a solution for waste management in the coffee industry, but also opens avenues for the production of valuable chemicals, aligning with sustainable development goals [26]. The biorefinery process is an example of a technology that uses hydrothermal treatment to extract valuable chemicals from lignocellulosic feedstocks. Various catalysts, such as mineral acids, ionic liquids, and metal oxides, have been studied for their ability to enhance the conversion of lignocellulose to sugars, formic acid, HMF, and other products [27]. For this reason, it is proposed to use organic and inorganic acids as catalysts and to analyze the impact of catalysis on the production of platform chemicals.

### 3.1. Without Catalyst

**Figure 3:**
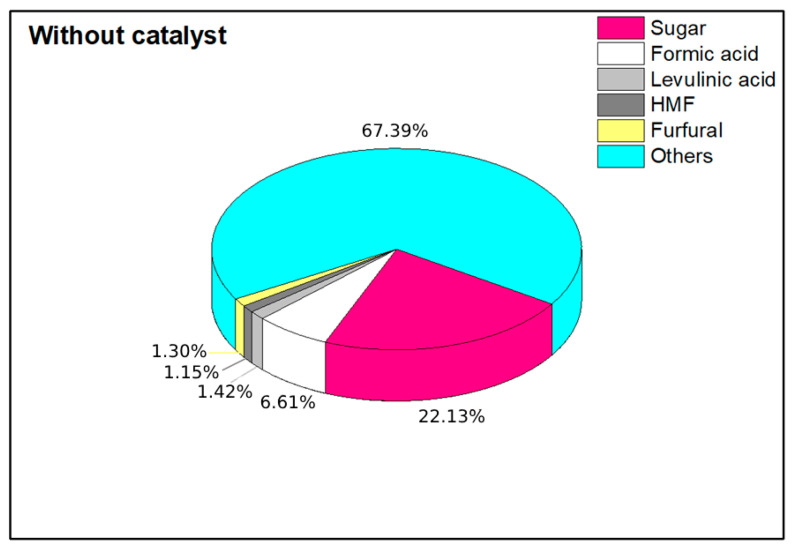
Obtention of platform chemicals by hydrothermal valorization of coffee cherry for 1 h at 180 °C without catalyst.

To provide a comparison, we conducted an experiment without the use of a catalyst. The experiment was performed at 180 °C for 1 h, using only water with a biomass–water ratio of 1:20. In Figure 3, the yields obtained under the previously mentioned conditions are presented.

The production results indicate that the total yield from the hydrothermal process was 32.60% without the use of a catalyst. Within this yield, the distribution of products was as follows: 22.13% was attributed to the production of sugars, which highlights the process’s effectiveness at selectively generating these valuable monomers. Additionally, 6.61% of the yield consisted of formic acid, while 1.42% was levulinic acid. The production of platform chemicals included 1.15% of 5-hydroxymethylfurfural (HMF) and 1.30% of furfural. Although the overall yield was not particularly high, the process demonstrated a strong selectivity for sugar production, making it a promising approach for generating key intermediates for further chemical transformations. This selectivity is crucial for applications in biorefineries, where maximizing the sugar yields can enhance the efficiency of downstream processes, such as fermentation and bioconversion into biofuels and other biochemicals.

**Figure 4:**
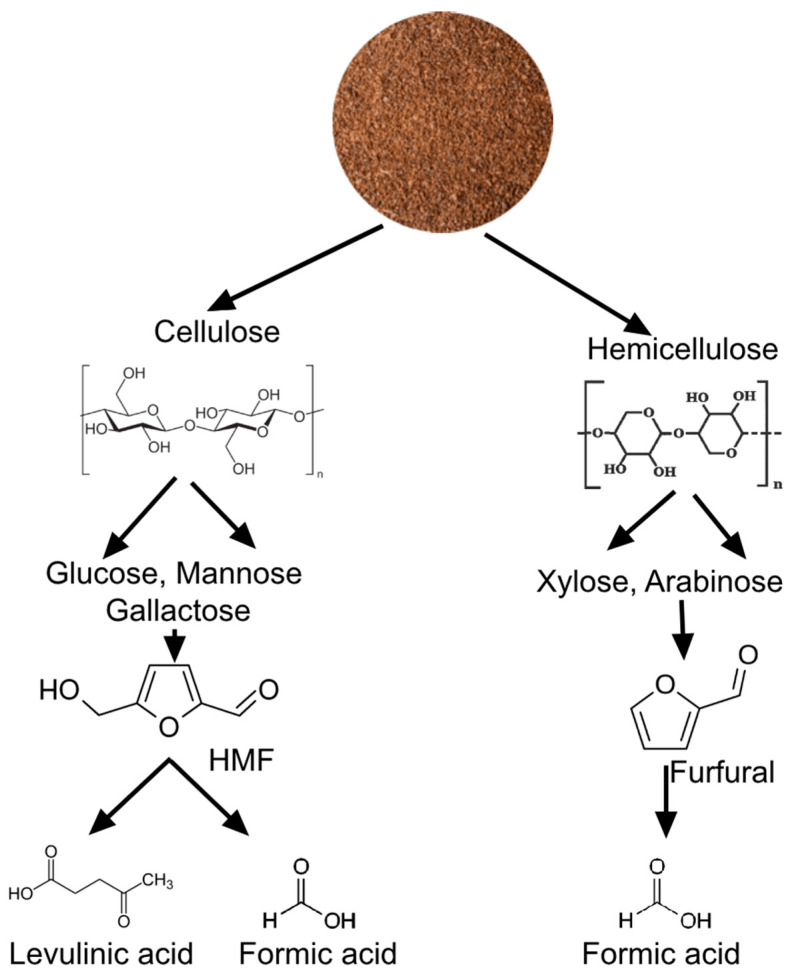
Specific products obtained and mechanism of obtention from coffee cherry waste.

The acid hydrolysis of cellulose and hemicellulose is a crucial step in the conversion of lignocellulosic biomass into valuable products (Figure 4). The mechanism involves the breakdown of these polysaccharides into their constituent monosaccharides, which can then undergo further transformations.

Cellulose, a homopolymer composed of glucose units linked by β-1,4-glycosidic bonds, is hydrolyzed under acidic conditions to release glucose monomers. This process is typically catalyzed by concentrated sulfuric acid at a moderate strength. The glucose monomers can then be further processed into 5-hydroxymethylfurfural (HMF) through dehydration reactions. HMF is a versatile platform chemical that can be converted into a variety of valuable products, such as levulinic acid and formic acid. Hemicellulose, a heterogeneous polymer consisting of various hexoses (glucose, mannose, and galactose) and pentoses (xylose and arabinose), undergoes acid hydrolysis to release these monomeric sugars. The pentoses, particularly xylose, can be dehydrated under acidic conditions to form furfural, another important platform chemical with numerous applications in the production of fuels, resins, and other chemicals.

Once hydrolyzed into monomeric sugars, these compounds can undergo further transformation into valuable chemical intermediates. For instance, glucose can be dehydrated to form 5-hydroxymethylfurfural (HMF) under acidic conditions, a critical precursor for biofuels and bioplastics. Similarly, xylose can be converted into furfural, another valuable platform chemical, through dehydration. Furthermore, HMF can be further processed into levulinic acid and formic acid, which have numerous applications in the chemical industry. The transformation of these sugars into furans and acids not only enhances the value of the biomass, but also contributes to the development of sustainable alternatives to fossil fuels and petrochemicals. The choice of hydrolysis and subsequent reaction conditions plays a crucial role in determining the yield and type of products obtained, highlighting the importance of optimizing these processes for efficient biomass utilization.

### 3.2. Results Obtained for Acid Catalysis

A table summing up all the information regarding the catalysts used is presented below (Table 2).

### 3.3. Effect of Inorganic Acids

Sulfuric acid is widely utilized in hydrothermal processes to valorize lignocellulosic biomass due to its strong catalytic properties that enhance the hydrolysis of complex carbohydrates [28]. The mechanism involves the protonation of hydroxyl groups in cellulose and hemicellulose, which facilitates the cleavage of glycosidic bonds, leading to the formation of simpler sugars, such as glucose and xylose. These sugars can then undergo further transformations to produce valuable platform chemicals [29]. Sulfuric acid’s effectiveness is particularly notable for its ability to operate under relatively mild conditions, which helps to minimize the degradation of sugars into by-products. The primary products yielded from this process include levulinic acid and furfural, both of which are important intermediates in the production of biofuels and biochemicals [30]. The use of sulfuric acid not only increases the overall yield of these platform chemicals, but also improves the efficiency and specificity of the hydrothermal conversion process, making it a vital component in the valorization of lignocellulosic biomass [28].

Hydrogen chloride (HCl) is utilized as a catalyst in the hydrothermal carbonization (HTC) of lignocellulosic biomass, enhancing the conversion efficiency and product yield [31]. In the HTC process, HCl acts by facilitating the hydrolysis and decomposition of complex biomacromolecules, which leads to the formation of various intermediates that can be further transformed into valuable products, such as hydrochar and bio-oil [18]. The presence of HCl lowers the activation energy required for these reactions, thus improving the overall effectiveness of the biomass conversion at relatively lower temperatures compared to non-catalytic processes [32]. Studies have demonstrated the successful application of HCl to HTC, for instance, in the valorization of municipal sludge and food waste digestate, where it significantly influenced the characteristics of the produced hydrochar and the recovery of nutrients like phosphorus [33]. The incorporation of HCl not only enhances the yield of solid and liquid products, but also contributes to the environmental sustainability of biomass treatment by reducing waste and recovering valuable resources.

Nitric acid is utilized as a catalyst in hydrothermal carbonization (HTC) to enhance the conversion of biomass into hydrochar, a carbon-rich solid material [34]. Acting as an acid catalyst, nitric acid facilitates hydrolysis and carbonization reactions, improving the carbon yields and modifying the properties of the resulting hydrochar. This treatment has been particularly effective with various feedstocks, such as sewage sludge, where it can enhance the energy potential and leach out undesirable metals, resulting in hydrochars with better combustion characteristics [35]. Previous studies have demonstrated its versatility, including applications in activated carbon functionalization and waste management strategies, highlighting nitric acid’s significant role in optimizing HTC processes for energy recovery and environmental sustainability [32].

Phosphoric acid is employed as a catalyst in hydrothermal carbonization (HTC) to enhance the conversion of biomass into valuable carbon-rich materials. The mechanism at work involves the acid facilitating the breakdown of complex organic structures, promoting hydrolysis and subsequent carbonization reactions [36]. This leads to increased yields of solid carbon products while improving the structural properties of the resulting hydrochar. For instance, studies have demonstrated that using phosphoric acid in the hydrothermal pretreatment of food waste significantly increases the concentration of soluble substances, such as proteins and carbohydrates, by approximately 65% compared to untreated biomass [33]. The optimal conditions for this process typically involve moderate temperatures and short reaction times, which contribute to energy efficiency and effective biomass conversion [35]. Previous research has highlighted the effectiveness of phosphoric acid for various biomass types, including food waste and agricultural residues [37]. In one study, the hydrothermal treatment of banana fibers with phosphoric acid resulted in activated carbons with enhanced porosity and electrochemical performance. The addition of phosphoric acid not only improved the yield of carbon products, but also facilitated the fixation of carbon atoms during the HTC process, leading to higher preparation yields than conventional methods [38]. This catalytic approach has shown promise for sustainable production processes, allowing for the recycling of phosphoric acid and reducing environmental impacts while maximizing the utility of the biomass waste [39].

The hydrothermal valorization of coffee cherry waste using various inorganic acid catalysts yielded distinct results, highlighting the influence of the catalyst type on the production of platform chemicals. The yields of sugars, formic acid, levulinic acid, HMF, and furfural were significantly affected by the choice of acid catalyst, demonstrating the importance of catalyst selection in optimizing this bioprocess (Table 2).

Sulfuric acid emerged as the most effective catalyst, achieving the highest total yield of 51.24%. This was primarily due to its ability to produce a substantial amount of sugars (40.33%) and HMF (2.67%), both of which are critical for subsequent chemical transformations. The high sugar yield indicates that sulfuric acid significantly enhances the hydrolysis of the cellulose and hemicellulose present in coffee cherry waste. In comparison, nitric acid yielded a total of 35.21%, with moderate sugar production (26.19%) and HMF (1.33%). While nitric acid performed well, it did not match the efficiency of sulfuric acid in promoting the hydrolysis process.

Hydrogen chloride, on the other hand, resulted in the lowest total yield at 28.88%. The sugar yield was only 20.49%, and HMF production was minimal at 0.76%. This suggests that hydrogen chloride may not be as effective in catalyzing the hydrolysis of biomass compared to stronger acids like sulfuric and nitric acids. Phosphoric acid also demonstrated a lower overall performance, with a total yield of 31.07%. Although it produced the highest yield of formic acid (6.99%), its sugar yield (21.57%) and lack of furfural production indicate limited effectiveness in promoting the full range of desired platform chemicals.

The selectivity of the catalysts varied significantly among the different acids. Phosphoric acid showed a strong preference for formic acid, which may be advantageous in applications where formic acid is a desired product. However, its overall yield was compromised due to the lower sugar and furfural production. The observed lack of furfural production with certain catalysts presents an intriguing aspect of this study that warrants further exploration, particularly regarding the potential chemical pathways influenced by these catalysts. Furfural is typically produced through the dehydration of pentose sugars derived from hemicellulose, and the efficiency of this reaction can be significantly affected by the acidity and structural characteristics of the catalysts used. For instance, catalysts that do not provide sufficient acidity or that stabilize the reaction intermediates may hinder the dehydration process, leading to lower yields of furfural. Additionally, the presence of competing reactions, such as the formation of other platform chemicals like HMF or levulinic acid, may divert the reaction pathways away from furfural production. In contrast, sulfuric acid not only provided high yields but also demonstrated a balanced production of multiple chemicals, contributing to its overall efficiency. The ability of sulfuric acid to facilitate both hydrolysis and subsequent dehydration reactions is likely responsible for its superior performance in producing a diverse array of platform chemicals.

The results underscore the impact of acid strength on the hydrothermal valorization process. Strong acids, like sulfuric acid, effectively lower the activation energy required for hydrolysis, leading to higher sugar yields. In contrast, weaker acids, such as hydrogen chloride, may not provide sufficient protonation to break down the complex polysaccharides effectively. This difference in acid strength directly correlates with the observed yields of the platform chemicals, emphasizing the need for the careful selection of catalysts based on their acidity and reactivity.

The findings from this study have significant implications for the sustainable production of chemicals from agricultural waste. The ability to convert coffee cherry residues into high-value products using sulfuric acid not only enhances the yield but also promotes the development of green technologies for biomass conversion. By maximizing the extraction of platform chemicals, this approach contributes to waste reduction and supports a circular economy in the agricultural sector.

### 3.4. Effect of Organic Acids

Chloroacetic acid, while not extensively studied as a catalyst in hydrothermal carbonization (HTC), possesses chemical properties that suggest it could be an effective acid catalyst for this process, primarily due to its stronger acidity compared to acetic acid, attributed to the electron-withdrawing effect of the chlorine atom [40]. This enhanced acidity may improve hydrolysis and dehydration reactions, facilitating the breakdown of complex organic structures and promoting the formation of smaller molecules [41]. However, the specific effects and advantages of chloroacetic acid in HTC remain largely unexplored, indicating a need for further research. Similarly, phthalic acid, a dicarboxylic acid, has not been specifically researched in HTC, but its structure suggests it could effectively donate protons, enhancing the hydrolysis and dehydration reactions necessary for converting complex organic materials into simpler compounds [42]. Benzoic acid, also lacking specific research in this context, could facilitate similar reactions due to its carboxylic acid nature and stable aromatic structure, potentially influencing reaction pathways and aiding in the formation of hydrochar and platform chemicals. Additionally, phenylacetic acid, known for its applications in pharmaceuticals and organic synthesis, showcases unique chemical properties that could enhance reaction efficiency in biocatalytic processes, making it a candidate for further exploration in biomass conversion applications. Overall, while these acids have not been thoroughly investigated in HTC, their chemical characteristics suggest potential roles as catalysts that warrant further study to assess their feasibility in biomass conversion processes.

Adipic acid, although not widely studied as a catalyst in hydrothermal carbonization (HTC), has the potential to enhance biomass conversion processes due to its dicarboxylic acid properties. As a proton donor, adipic acid could facilitate hydrolysis and dehydration reactions during HTC, similar to other acid catalysts, potentially breaking down complex organic structures in biomass and increasing the yield of hydrochar and platform chemicals [43]. While direct evidence of its application in HTC is limited, the general principles of acid catalysis suggest that adipic acid could improve the efficiency of biomass conversion by promoting the formation of valuable chemicals, such as sugars, furfurals, and organic acids. Acetic acid, on the other hand, has been utilized as a catalyst in HTC to enhance the conversion of biomass into hydrochar and platform chemicals, primarily due to its acidic properties that facilitate hydrolysis and dehydration reactions [44]. Studies have shown that acetic acid can increase the high heating value of hydrochar and improve the overall efficiency of biomass conversion, leading to higher yields of carbon-rich materials [45]. Acetic acid has been effective for various biomass types, including woody biomass and agricultural residues, and its combination with other catalysts, such as sodium hydroxide, has been explored to optimize biomass conversion processes [46]. Butyric acid has also never been investigated as a catalyst in HTC.

Limited research has been conducted on the use of anthranilic acid, propanoic acid, aminobenzoic acid, and succinic acid as catalysts in hydrothermal carbonization (HTC), but their chemical properties suggest potential benefits for enhancing biomass conversion processes. Anthranilic acid, an aromatic amine with a carboxylic acid group, could theoretically facilitate hydrolysis and dehydration reactions during HTC, potentially increasing the yield of hydrochar and valuable platform chemicals, although no direct studies have been documented. Similarly, propanoic acid, a medium-chain fatty acid, may enhance these critical reactions due to its carboxylic acid characteristics, leading to improved yields of hydrochar and other products. Aminobenzoic acid, which possesses both an amine and a carboxylic acid functional group, could act as a weak acid catalyst, influencing the breakdown of complex organic materials in biomass. Lastly, succinic acid, a dicarboxylic acid, could provide the necessary acidity to promote hydrolysis and dehydration reactions, thereby enhancing the conversion of biomass into fermentable sugars and valuable chemicals like HMF and levulinic acid. Overall, while specific applications of these acids in HTC are not well-documented, their structural properties indicate they could play significant roles in improving biomass conversion efficiency.

The analysis of various organic acids as catalysts in the hydrothermal carbonization (HTC) process reveals significant differences in their effectiveness and influence on reaction mechanisms (Table 2). The data indicate that the total yields of platform chemicals vary considerably among the acids tested, with adipic acid achieving the highest total yield of 53.66%. This suggests that its dicarboxylic acid structure may enhance both hydrolysis and dehydration reactions, crucial for breaking down a complex biomass into simpler compounds. In contrast, butyric acid exhibits the lowest total yield at 17.4%, highlighting its limited efficiency in promoting the necessary reactions during HTC. The differences in yields underscore the importance of selecting the appropriate acid catalyst to optimize biomass conversion.

The functional groups present in organic acids play a critical role in determining their catalytic efficiency [47]. Acidic functional groups, such as carboxylic acid groups, are essential for facilitating hydrolysis and dehydration reactions. For instance, benzoic acid, with its aromatic structure and carboxylic acid group, achieved a total yield of 41.15%, indicating its effectiveness in stabilizing reaction intermediates and promoting the breakdown of lignocellulosic biomass. Similarly, chloroacetic acid, despite a slightly lower total yield of 31.88%, demonstrated the potential to influence reaction pathways due to the electron-withdrawing effect of the chlorine atom, which may enhance the acid’s proton-donating ability. This highlights how the presence of specific functional groups can enhance the selectivity and efficiency of catalysts in HTC.

Selectivity towards specific platform chemicals also varies among the acids, impacting the overall efficiency of the HTC process. Adipic acid not only provided the highest total yield, but also showed remarkable selectivity for formic acid (19.66%) and levulinic acid (11.29%), suggesting its dual role in hydrolysis and subsequent reactions. In contrast, butyric acid did not yield any formic or levulinic acid, indicating a lack of selectivity and efficiency in producing valuable chemicals. The ability of a catalyst to influence the production of specific platform chemicals is crucial for tailoring the HTC process to produce the desired outcomes, such as maximizing sugar or organic acid yields for further applications.

The mechanism of the HTC process is also influenced by the type of acid catalyst used. Stronger acids, such as adipic and benzoic acids, likely enhance the cleavage of glycosidic bonds in polysaccharides, resulting in higher sugar yields. For instance, benzoic acid achieved a sugar yield of 25.48%, which is significant compared to the other acids. In contrast, propanoic acid and phenylacetic acid showed moderate yields of sugars (19.78% and 21.71%, respectively), suggesting that their medium-chain structures may not be as effective in promoting the hydrolysis of a complex biomass. This illustrates the importance of acid strength and structure in determining the efficiency of the HTC process.

Furthermore, the presence of multiple functional groups within certain acids can influence the reaction pathways and product distributions. Aminobenzoic acid, which contains both amine and carboxylic acid functional groups, could theoretically facilitate hydrolysis and dehydration reactions, although its total yield of 26.07% indicates limited effectiveness in practice. This suggests that while the presence of diverse functional groups can provide catalytic advantages, the overall structure and acidity of the acid are critical factors that determine the success of the HTC process.

This analysis of various organic acids as catalysts for hydrothermal carbonization highlights the significant role that acid structure and functional groups play in influencing the reaction mechanisms, selectivity, and overall efficiency. Adipic acid stands out as the most effective catalyst, achieving the highest total yield and selectivity for valuable platform chemicals. In contrast, acids like butyric and propanoic acid demonstrate lower yields and selectivity, indicating their limited utility in HTC. These findings emphasize the importance of selecting appropriate acid catalysts to optimize biomass conversion processes and enhance the production of high-value chemicals and hydrochar. Future research should focus on exploring additional organic acids and their combinations to further improve the efficiency and selectivity of the HTC process, potentially leading to more sustainable and economically viable biomass conversion technologies.

### 3.5. Organic Acids vs. Inorganic Acids

The hydrothermal carbonization (HTC) process aims to convert biomass into valuable platform chemicals, and the choice of catalyst plays a crucial role in determining the efficiency and selectivity of this process. The comparison of organic and inorganic acid catalysts reveals that both categories can significantly enhance the yields of sugars, formic acid, levulinic acid, HMF, and furfural. However, the effectiveness of each catalyst varies, depending on its specific properties and the desired outcome of the HTC process.

Among the inorganic acid catalysts, sulfuric acid stands out as the most effective, achieving a total yield of 51.24%. This can be attributed to its strong acidity, which facilitates the hydrolysis of cellulose and hemicellulose in biomass, leading to a substantial sugar yield of 40.33% and a notable production of HMF of 2.67%. The high sugar yield suggests that sulfuric acid efficiently breaks down complex polysaccharides into simpler compounds, making them available for further conversion into valuable chemicals. In contrast, hydrogen chloride results in the lowest total yield of 28.88%, indicating its limited effectiveness in promoting the necessary reactions for biomass conversion.

Organic acids also show promising results, with adipic acid achieving the highest total yield of 53.66%, surpassing even sulfuric acid. Adipic acid’s remarkable selectivity for formic acid (19.66%) and levulinic acid (11.29%) suggests its dual role in facilitating both hydrolysis and subsequent reactions. The presence of multiple carboxylic acid groups in adipic acid may contribute to its high selectivity for these specific platform chemicals. Benzoic acid also performs well, yielding 41.15%, likely due to its aromatic structure, which may stabilize reaction intermediates and enhance the breakdown of complex organic materials. However, some organic acids, such as butyric acid, show limited effectiveness, yielding only 17.4%, highlighting the variability in performance among the different organic acids.

The differences in yields among the catalysts can be attributed to their acidity, functional groups, and ability to promote specific reactions. Strong acids, like sulfuric and adipic acids, likely enhance the cleavage of glycosidic bonds in polysaccharides, resulting in higher sugar yields. Organic acids with aromatic structures, such as benzoic acid, may provide additional stability and influence the reaction pathways, potentially enhancing the breakdown of complex organic materials. The electron-withdrawing effect of the chlorine atom in chloroacetic acid may improve its proton-donating ability, leading to its moderate performance.

The environmental and economic implications of the findings of this study are substantial, particularly regarding the scaling up of hydrothermal carbonization (HTC) for industrial applications. By converting agricultural waste, such as coffee cherry waste, into high-value platform chemicals, the HTC process promotes a circular economy and reduces reliance on fossil fuels, thereby minimizing greenhouse gas emissions and waste generation. This sustainable approach enhances waste management by diverting organic materials from landfills and incineration, which not only mitigates their environmental impacts but also conserves valuable resources. The production of hydrochar as a renewable energy source further contributes to the environmental benefits of the HTC process, supporting the transition towards more sustainable energy systems.

Economically, the efficient conversion of agricultural waste into high-value products can create new revenue streams and job opportunities, particularly in rural areas where such waste is abundant. The scalability of the HTC process is crucial for its economic feasibility, and optimizing catalyst selection is a key factor in ensuring the process’s efficiency and cost effectiveness. Effective catalysts, such as sulfuric and adipic acids, can significantly enhance yields and reduce operational costs, making HTC a more attractive alternative to traditional chemical synthesis routes. Additionally, integrating HTC with other valorization pathways, like anaerobic digestion or pyrolysis, can maximize resource recovery and minimize waste streams, further enhancing the overall economic and environmental performance of biomass conversion technologies in industrial settings.

## 4. Conclusions

This study presents a comprehensive method for utilizing both organic and inorganic acid catalysts (0.02 M) in hydrothermal carbonization (HTC), at 180 °C for 1 h in a batch reactor, a process aimed at converting coffee cherry biomass (in a 1:20 ratio) into valuable platform chemicals. The findings underscore the critical role that catalyst selection plays in optimizing the yields and selectivity of the desired products. Among the catalysts tested, adipic acid achieved the highest total yield of 53.66%, demonstrating its effectiveness in promoting the production of formic acid and levulinic acid. In contrast, butyric acid produced the lowest total yield of 17.4%, highlighting its limited utility in enhancing the HTC process.

The results indicate that inorganic acids can also significantly enhance biomass conversion, particularly sulfuric acid, which achieved a total yield of 51.24%. This suggests that strong acids are effective at facilitating hydrolysis and the subsequent reactions necessary for breaking down complex organic structures. However, the performance of organic acids, like adipic and benzoic acids, illustrates that they can be equally or more effective in certain contexts, especially when targeting specific platform chemicals. The ability of these acids to stabilize reaction intermediates and influence reaction pathways is crucial for improving the overall efficiency of the HTC process.

This study establishes a clear framework for employing organic and inorganic acid catalysts in hydrothermal carbonization, showcasing the potential for significant improvements in the yield and selectivity of platform chemicals. The contrasting performances of the different catalysts emphasize the necessity of tailored approaches to catalyst selection, based on the specific biomass feedstock and desired end products. This research sets the stage for future investigations aimed at refining these methods and expanding their applications in the field of biomass conversion.

The findings from this study have significant implications for potential industrial applications, as they demonstrate the ability to convert coffee cherry waste into valuable platform chemicals through hydrothermal carbonization. The production of key chemicals, such as sugars, HMF, furfural, levulinic acid, and formic acid, from readily available agricultural waste presents new opportunities for sustainable and profitable industrial processes. By optimizing catalyst selection, particularly with effective options like sulfuric acid and adipic acid, the HTC process can be scaled up for large-scale operations, making it an attractive solution for industries aiming to adopt greener technologies and reduce their environmental impact while maximizing resource recovery.

## Figures and Tables

**Table 1:** Characterization of coffee waste [24].

Essay	Coffee Cherry (%)
Dry matter (%)	95.5
Hemicellulose	12.5
Lignin	13.7
Cellulose	27.6
C	45.27
H	4.862
N	1.471
S	0.138
Moisture (initial biomass)	80.79
Moisture (BHP) **	10.94
Ashes	7.79
Volatile matter	10.06
Fixed carbon	1.36

** Biomass for hydrothermal processes.

**Table 2:** Results obtained for hydrothermal valorization of coffee cherry waste using organic and inorganic catalysts.

Catalyst	Sugars (%)	Formic Acid (%)	Levulinic Acid (%)	HMF (%)	Furfural (%)	Others (%)	Total Yield (%)
Without	22.13	6.61	1.42	1.15	1.3	67.39	32.61
Sulfuric acid	40.33	5.4	0.64	2.67	2.2	48.76	51.24
Hydrogen chloride	20.49	5.92	1.11	0.76	0.6	71.12	28.88
Nitric acid	26.19	5.82	0.99	1.33	0.88	64.79	35.21
Phosphoric acid	21.57	6.99	1.28	1.23	0	68.93	31.07
Chloroacetic acid	23.21	4.47	1.11	1.76	1.33	68.12	31.88
Phtalic acid	19.58	4.22	0.94	1.59	1.25	72.42	27.58
Benzoic acid	25.48	9.85	3.28	1.42	1.12	58.85	41.15
Phenylacetic acid	21.71	5.19	1.28	1.3	11.46	59.06	40.94
Adipic acid	20.59	19.66	11.29	1.18	0.94	46.34	53.66
Acetic acid	20.94	11.12	1.33	1.22	0.84	64.55	35.45
Butyric acid	16.79	0	0	0.38	0.23	82.60	17.4
Anthranilic acid	19.47	5.7	1.42	0.89	0.57	71.95	28.05
Propanoic acid	19.78	3.56	2.44	1.93	0	72.29	27.71
4-aminobenzoic acid	19.27	4.69	1.22	0.36	0.53	73.93	26.07
Succinic acid	21.49	5.21	1.39	1.37	1.06	69.48	30.52

## Data Availability

All the data are presented in this manuscript.
